# Progress in the therapeutic inhibition of Cdc42 signalling

**DOI:** 10.1042/BST20210112

**Published:** 2021-06-08

**Authors:** Natasha P. Murphy, Helen R. Mott, Darerca Owen

**Affiliations:** Department of Biochemistry, University of Cambridge, 80 Tennis Court Road, Cambridge CB2 1GA, U.K.

## Abstract

Cdc42 is a member of the Rho family of small GTPases and a key regulator of the actin cytoskeleton, controlling cell motility, polarity and cell cycle progression. It signals downstream of the master regulator Ras and is essential for cell transformation by this potent oncogene. Overexpression of Cdc42 is observed in several cancers, where it is linked to poor prognosis. As a regulator of both cell architecture and motility, deregulation of Cdc42 is also linked to tumour metastasis. Like Ras, Cdc42 and other components of the signalling pathways it controls represent important potential targets for cancer therapeutics. In this review, we consider the progress that has been made targeting Cdc42, its regulators and effectors, including new modalities and new approaches to inhibition. Strategies under consideration include inhibition of lipid modification, modulation of Cdc42–GEF, Cdc42–GDI and Cdc42-effector interactions, and direct inhibition of downstream effectors.

## Introduction

The human Ras superfamily consists of 167 members which subdivide into five families of small GTPases, with conserved structures and highly related regulatory mechanisms. Their capacity to bind to guanine nucleotides underpins their ability to act as binary molecular switches, controlling multiple signalling pathways and supervising numerous cellular functions. The Rho family is one branch of the Ras superfamily.

In the resting state Rho family GTPases are GDP-bound but nucleotide exchange facilitates binding of GTP, resulting in conformational changes that allow the GTPase to interact with its immediate downstream effector proteins, triggering signalling cascades. GTP hydrolysis returns the G protein switch to its GDP-bound resting state. The processes of nucleotide exchange and hydrolysis for most small G proteins are extremely inefficient and therefore the GTPases require regulatory proteins to function successfully. Guanine nucleotide exchange factors (GEFs) promote GTP binding and activate the GTPases, while GTPase activating proteins (GAPs) increase GTP hydrolysis and therefore switch off the small G proteins. Rho family GTPases are also regulated by the RhoGDI proteins, which have a number of regulatory roles. They act as negative regulators by inhibiting nucleotide exchange and sequestering the inactive G proteins in the cytosol. However, they also act as chaperones, protecting the small G proteins from degradation and facilitating their localization to the correct cellular membrane.

The Rho family protein Cdc42 is weakly transforming in its own right but essential for transformation by Ras, the most highly mutated oncogene found in human cancer [1,2]. Deletion of Cdc42 from Ras-transformed cells results in a decrease in cell cycle progression and therefore cell proliferation [3]. Likewise, overexpression of Cdc42 is observed in several cancers, where it is linked to poor prognosis [4]. Like many of the Rho family GTPases, Cdc42 is a key regulator of the actin cytoskeleton and therefore controls both cell architecture and motility. Deregulation of Cdc42 is also therefore linked to tumour metastasis [5,6]. Mutations in Cdc42 itself are rarely found in cancers, however alterations to its regulators have been extensively characterized with many GEFs identified as oncogenes [7] and some GAPs as tumour suppressors [8]. Cdc42 and other components of its signalling pathways therefore represent potential targets for cancer therapeutics. We have explored the subversion of Cdc42 regulated signalling in cancer in a recent review [9]. Further investigation into the specific mechanisms by which these alterations drive cancers will be important in informing therapeutic strategies, as are now being considered for targeting Ras signalling [10].

**Figure 1.**
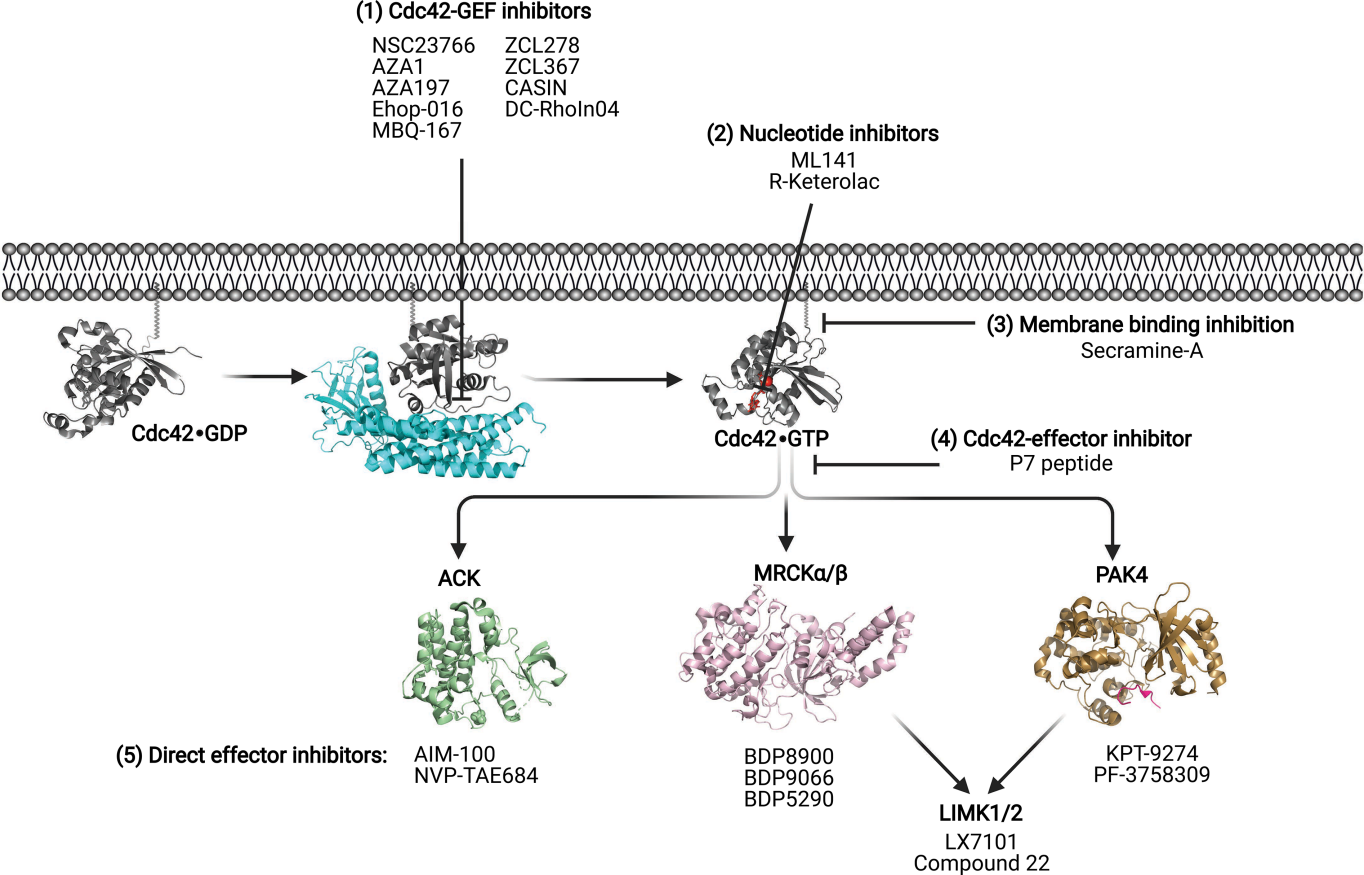
Different approaches to inhibition of Cdc42. (**1**) Cdc42–GEF small molecule inhibitors: ZCL278, ZCL367, CASIN, NSC23766, AZA1, AZA197, Ehop-016, MBQ-167 and DC-Rhoin04 (**2**) Cdc42 nucleotide binding inhibitors: ML141, R-Ketorolac (**3**) Membrane binding inhibitors: Secramine-A (**4**) Cdc42-effector complex inhibitor: P7 peptide (**5**) Effector inhibitors: ACK (AIM-100, NVP-TAE684), MRCK (BDP8900, BDP9066, BDP5290), LIMK1/2 (LX7101) and PAK4 (KPT-9274, PF-3758309). PDB structures shown: Cdc42·GDP (PDB:1DOA), Cdc42 in complex with GEF region of Intersectin (PDB: 1KI1), Cdc42·GTP with GMPPNP nucleotide shown in red (PDB:1NF3), ACK1 kinase domain (PDB: 4HZR), MRCKα (PDB: 4AW2), PAK4 kinase domain in complex with LIMK1 substrate peptide (PDB: 6WLY).

Targeting Rho family-controlled signalling pathways is an approach that has recently enjoyed increased attention within the cancer therapeutics field, with strategies to target Cdc42 also having potential applications in neurodegeneration [11–13] and infectious diseases [14]. There are multiple ways in which inhibition of Cdc42 controlled pathways has been approached and these are summarized in Figure 1. Strategies include inhibition of lipid modification, inhibition of Cdc42-regulator and effector interactions, direct inhibition of effector kinases and covalent irreversible inhibition of GEF-catalyzed nucleotide exchange. These schemes, along with future potential approaches are reviewed here.

## Therapeutic strategies

### Targeting nucleotide binding of Cdc42

Given that the activity of Rho GTPases is governed by the nucleotide they are bound to, inhibiting GTP binding is an obvious inhibition strategy. The challenges for generating molecules that can target the nucleotide binding pockets of small G proteins and outcompete the picomolar binding affinity of the nucleotide are well documented [15]. Nevertheless, some small molecules have been discovered which employ this approach. ML141 (Figure 1) was identified in a high throughput screen against a panel of GTPases representing key members of the Ras superfamily, for its ability to decrease GTP binding to Cdc42 [14]. It was suggested to bind to an allosteric site of Cdc42 to induce dissociation of the nucleotide. However, ML141 does not inhibit cell migration, a readout for active Cdc42, or suppress Cdc42 activity [16]. Similarly the R-enantiomer of the small molecule Ketorolac, acts by allosteric inhibition of nucleotide binding to Cdc42 and its close relative Rac1 [17]. Ovarian epithelial cancer cell lines treated with R-Ketorolac however showed reduced migration and invasion and a block in both PAK1 and PAK2 signalling (effectors of Cdc42 and Rac1) [18].

### Targeting Cdc42–GEF interactions

One of the central approaches to targeting Cdc42 involves direct targeting of the interfaces of specific Cdc42–GEF complexes and a number of small molecule inhibitors have been identified (Figure 1, reviewed recently in [19,20]). The major challenges facing inhibition of specific Cdc42–GEF interactions are the overlapping interfaces by which GEFs bind the GTPase and the promiscuous activity of the GEFs towards Rho family members [21,22], creating problems of achieving specificity coupled with efficacy. Many of the molecules which have been developed presently therefore are pan or at least dual Rho family GTPase inhibitors of both Cdc42 and Rac1. There is however the possibility of developing more specific inhibitors as more detailed knowledge of the complexes becomes available. In terms of pan Rho family inhibitors, it can also be argued that a more promiscuous drug may be more beneficial for reducing resistance to a therapy against a single individual Rho GTPase target, especially as many of the cellular functions of Rho family GTPases rely on the fine balance between several family members [23]. On the other hand, dissecting and assessing the individual biological effects due to inhibition of Cdc42 demands specificity and selectivity within the Rho family proteins.

#### Targeting Cdc42–GEF interfaces

**Figure 2.**
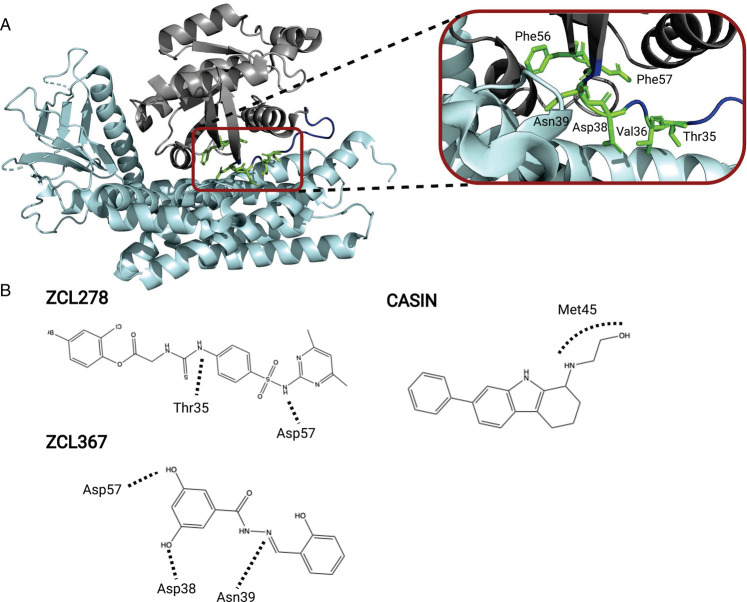
Inhibition of Cdc42-Intersectin GEF interaction. (**A**) Cdc42 (grey) in complex with GEF Intersectin (cyan) (PDB:1KI1) with Switch I of Cdc42 (dark blue) and key interacting residues for small molecule inhibitors ZCL278 and ZCL376 (green); Thr35, Val36, Asp38, Asn39, Phe56 and Asp57 (**B**) Schematic representations of interactions between Cdc42 residues and molecules ZCL278, ZCL367 and CASIN. Interactions of ZCL278 include 2 hydrogen bonds with Thr35 and Asp57 and hydrophobic interactions with Thr35 and Val36. Interactions of ZCL367 involve 3 hydrogen bonds with Asp38, Asn39 and Asp57 and 2 hydrophobic interactions with Phe56 and Val36 of Cdc42. Interaction of CASIN involves a critical interaction with Met45 of Cdc42.

The complex which has been targeted most frequently is Cdc42-Intersectin (ITSN1). ITSN1 has been identified as a Cdc42 specific GEF in *in vitro* studies [24,25] and is one of several RhoGEFs showing a high frequency of alterations in some cancer types [26,27]. The small molecule ZCL278 was identified by a high throughput virtual screen where candidates were docked into the ITSN1 binding groove of Cdc42 (Figure 2). ZCL278 was predicted to make hydrogen bonds with Thr35, Asn39 and Asp57 of Cdc42 as well as hydrophobic interactions with Val36 and Phe56. ZCL278 bound Cdc42 with low micromolar affinity and inhibited multiple Cdc42-dependent cellular processes [28]. Despite its inhibitory effects, ZCL278 was however later shown to be a partial Cdc42 agonist under certain conditions and further work focussed on ZCL367, which possessed increased potency and greater selectivity in a further screen assaying A549 and PC3 cell migration [29]. ZCL367 was shown to have selective potency towards Cdc42 (IC_50_ = 0.098 µM) over RhoA (IC_50_ = 29.7 µM) and, albeit less so, Rac1 (IC_50_ = 0.19 µM). Wider screening of ZCL367 across a panel of lung and prostate cancer cell lines representing both EGFR- and Ras-driven cancers demonstrated inhibition of migration and proliferation [29]. Additionally, a lung cancer xenograft mouse model showed reduced tumour growth after treatment with ZCL367 and cell lines had reduced Cdc42-mediated filopodia formation [29].

Another small molecule, CASIN, binds to Cdc42·GDP with an affinity of ∼300 nM and inhibits the Cdc42-Intersectin catalyzed dissociation of GDP [30,31]. Docking indicated that CASIN binds Cdc42 at a site proximal to switch I where Met45 would form a critical interaction: mutation of Met45 (M45E) abrogated the ability of CASIN to bind Cdc42. CASIN has been demonstrated to inhibit F-actin mobilization and directional migration of cells. A study using drug-resistant models of multiple melanoma highlighted an application for CASIN and made progress in addressing some of potential side-effects of a Cdc42 inhibitor [31]. Most recently, CASIN has been found to extend murine lifespan by four days, highlighting an interesting alternative avenue of Cdc42 pharmacological inhibition in age-related applications [32].

**Figure 3.**
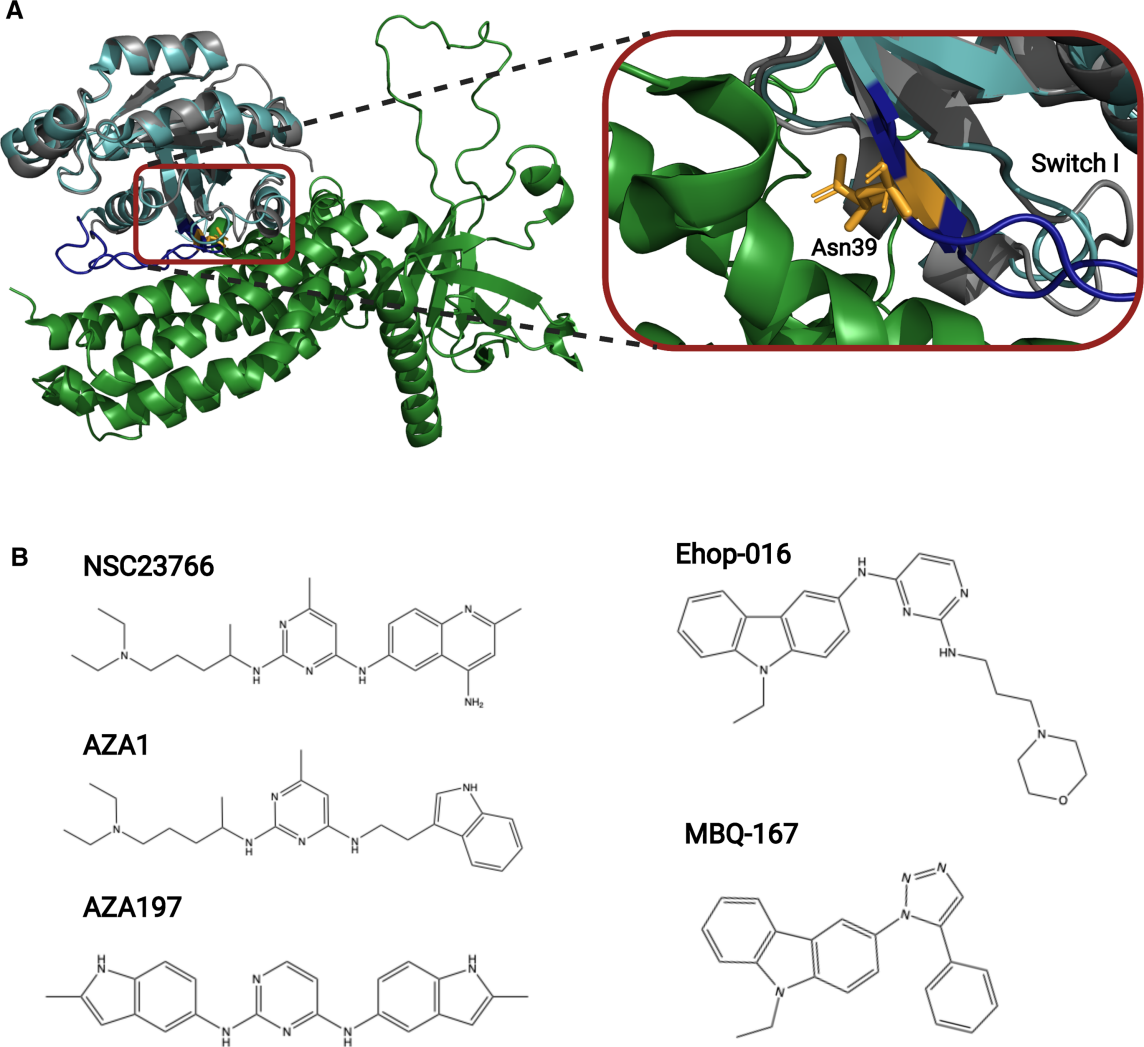
Inhibition of Cdc42/Rac-1-GEF interactions. (**A**) Structure of Rac1 (grey) in complex with Tiam1 (green) (PDB 1FOE). Cdc42 (cyan, PDB: 1NF3 is shown superimposed on Rac1. Asn39 (orange) located in switch I (dark blue) is shown as sticks, this residue is critical for the interaction with Ehop-016 and MBQ-167 (**B**) Schematic representation of small molecule inhibitors NSC23766, Ehop-016, MBQ-167, AZA1 and AZA197. NSC23766 was a founder small molecule for the development of Ehop-016. Ehop-016 was subsequently further matured into MBQ-167. NSC23766 was also a starting point molecule for the development of AZA1andAZA197.

In the development of Cdc42 inhibitors, targeting of its closest relative Rac1 has also been beneficial. NSC23766 [33] (Figure 3) is a founder molecule originally developed to inhibit the Rac1-Trio and Rac1-Tiam1 interfaces. NSC23766 was identified by virtual screening as a molecule that bound to the GEF-recognition groove of Rac1, centering on Trp56. Ehop-016 [34] was developed from NSC23766 [34], with both molecules containing a central 6-membered pyrimidine ring (Figure 3). As well as inhibiting Rac1 at an affinity of 1.1 µM, Ehop-016 was also found to inhibit Cdc42 activity at a concentration of 10 µM [35]. The same group also developed MBQ-167 using *in silico* modelling to identify a molecule capable of binding deeper into the NSC23766 binding groove identified on Rac1 [33], by exploiting the formation of a H-bond with Asn39. Asn39 is present in switch 1 of both Rac1 and Cdc42, hence MBQ-167 is a dual Cdc42/Rac1 inhibitor (Rac1, IC_50_ 103 nM; Cdc42, IC_50_ 78 nM). A 1,2,3-triazole ring in MBQ-167 replaces the central pyrimidine ring of Ehop-016, with a phenyl group as an ortho-substitution on the triazole ring. Characterization of MBQ-167 is one of only a few studies to take a Cdc42 inhibitor into a preclinical mouse model, where it inhibited HER-2 type tumour growth and metastasis in immunocompromised mice by 90% [36] The pharmacokinetic profiles of the small molecule were also evaluated, with relative bioavailability reported to be 35% with an oral half-life of 2.6 hours [37].

The Cdc42–Dbs interaction has also been targeted using molecules elaborated from NSC23766 [33]. AZA1 (Figure 3) was identified as a potential Rac1–GEF inhibitor based on NSC23766, in a virtual screen using the ZINC database [38]. AZA1 was shown to be a dual inhibitor for Rac1 and Cdc42, suppressing the activity of both Rho GTPases [39]. AZA1 has relatively low potency, which may be explained by the presence of many flexible bonds within the molecule and consequently the likely entropic penalty upon binding. A more constrained molecule, AZA197, has now been developed (Figure 3), which occupies the classic ‘flat’ small molecule chemical space [40]. Targeting the Cdc42–Dbs interaction resulted in good specificity, with no inhibition of Rac1 or RhoA reported and with nucleotide exchange on Cdc42 reduced by 61%. No structural data is available presently, so there are no details of the mode of binding of AZA197 to Cdc42 or how specificity is achieved. AZA197 inhibited proliferation, cell migration and invasion in two colorectal cancer cell lines, HT-29 and SW620. A murine xenograft model using SW620 cells showed reduced tumour growth and increased mouse survival *in vivo* [40].

Overall, an inhibitor of a GEF–Cdc42 interface would be an elegant therapeutic strategy to limit the pool of active Cdc42·GTP. However, specificity is important and has been highlighted and to some extent addressed through the studies discussed above. The challenge of specificity however raises an interesting avenue utilizing dual inhibition of Rac1 and Cdc42 as illustrated with MBQ-167.

Targeting RhoGEF activity itself is currently unexplored, although proof-of-concept has been demonstrated by the natural product brefeldin A which binds Arf1·GDP-exchange factor complexes, locking them into a conformation unable to proceed to nucleotide exchange [41]. It may also be possible to stabilize GEF-GTPase inactive complexes by displacement of the bound GDP molecule resulting in a stable non-productive complex. A fragment soaking approach using an XChem fragment library with crystals of the Rho GEF Kalirin complexed with Rac1·GDP, identified a fragment capable of displacing GDP by binding to the nucleotide-binding pocket. The original hit fragment, Z56880342, was used to generate ten further analogues which all bind in the GDP binding site of Rac1 in complex with Kalirin (PDB 5QQD) and represent starting points for further chemical optimization [42]. Hence, targeting GEF–Cdc42 complexes rather than direct inhibition of the GEFs themselves is another potential avenue for Cdc42 inhibition.

#### Irreversible cysteine-targeted inhibitors

A distinctly different method of inhibiting Cdc42–GEF interactions exploits a cysteine located spatially close to but not directly within the interface of the complex, to inhibit GEF-catalyzed nucleotide exchange. A set of covalent, irreversible inhibitor molecules have recently been developed targeting Cys107 of RhoA [43]. The tightest binder, DC-Rhoin04, inhibited nucleotide exchange on RhoA with a IC_50_ of ∼3 µM [43]. The screened molecules all contain electron-deficient alkenes capable of reacting with the nucleophilic cysteine of interest. The targeted cysteine is only present in Rho family small G proteins and hence provides selectivity over the remaining Ras superfamily proteins. As well as inhibiting the interaction between RhoA-LARG, when tested for selectivity it also inhibited the interaction between Cdc42-Intersectin and Rac1-Tiam1.

**Figure 4.**
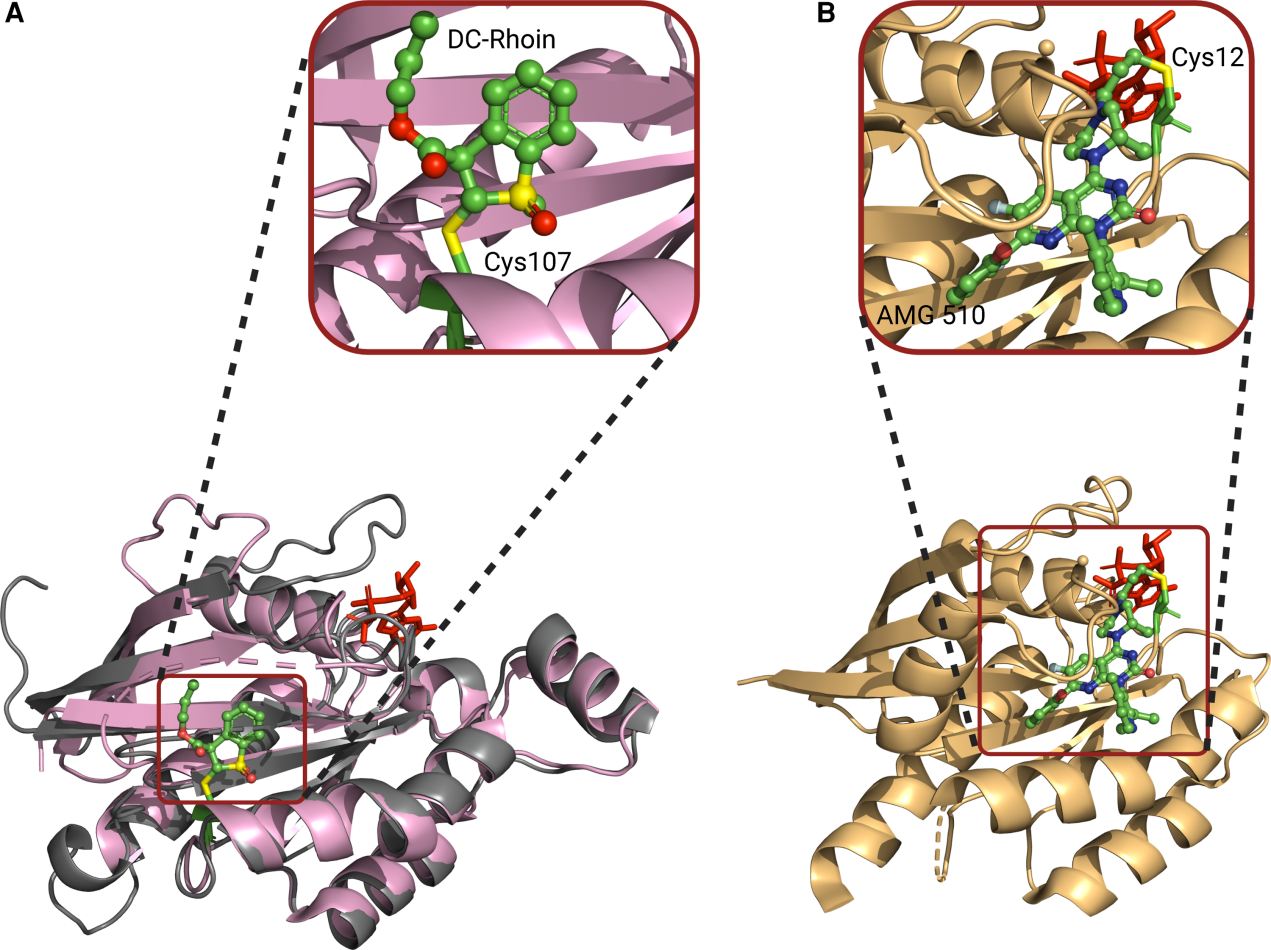
An irreversible cysteine-targeting covalent inhibitor of Cdc42. (**A**) Structure of DC-Rhoin04 bound to Cys107 (red) of RhoA (pink) (PDB 6KX3). Cdc42 (grey) displayed aligned to RhoA. (**B**) Crystal structure of K-Ras^G12C^ in complex with cysteine covalent inhibitor AMG 510 (PDB 6OIM), where AMG 510 binds to the switch II pocket of K-Ras^G12C^ in its inactive, GDP form exploiting a cryptic pocket formed by His95, Tyr96 and Gln99.

The co-crystal structure of DC-Rhoin04-RhoA revealed a pocket, termed the CLocK pocket, which extends out from Cys107 and is induced by DC-Rhoin04 binding. Cys105 in Cdc42 is the presumed site of action for DC-Rhoin04, allowing it to inhibit the Cdc42-Intersectin interaction (Figure 4). DC-Rhoin04 was shown to suppress migration and invasion in the breast cancer cell line MDA-MB-231. Due to the pan-specificity of the inhibitor it is not known through which Rho GTPase these effects are mediated, although it is likely that all are implicated.

Cysteine targeting covalent inhibitors have the potential to cross-react with exposed surface cysteines of off-target proteins and exhibit immunotoxicity where the inhibitor is highly reactive or lacks specificity [44]. The off-target potential cross-reactivity of the parent compound DC-Rhoin was investigated and it had no activity against Ras family GTPases, which lack a Cys105/107 equivalent, or ten epigenetic targets with exposed cysteines [43]. The duration of target inhibition by DC-Rhoin is yet to be investigated and this parameter is often critical to their success, since toxicities can be associated with extended inhibition [45].

This strategy of developing an irreversible inhibitor targeting a cysteine residue has most notably been successfully applied to targeting G12C mutations of K-Ras [46], where compounds MRTX849 and AMG-512 are currently in clinical trials [47]. Whilst these compounds target an oncogenic cysteine mutation in GDP-bound, inactive K-Ras [46,48] (Figure 4), the DC-Rhoin molecules targets Cys105/107 in wild type RhoA/Cdc42. DC-Rhoin binds the GDP-bound and apo forms and may well bind the GTP form as the cysteine is solvent exposed there too. DC-Rhoin binds at the CLocK pocket that is adjacent to, but unique from, the pocket targeted in K-Ras^G12C^ (Figure 4). Given that the cancer associated mutations in Cdc42 are not likely to predominantly affect deactivation mechanisms, as is found for K-Ras [10], selectivity for Cdc42 in both nucleotide forms may have therapeutic relevance.

### Targeting Cdc42-effector interactions

#### Targeting Cdc42-effector interfaces

**Figure 5.**
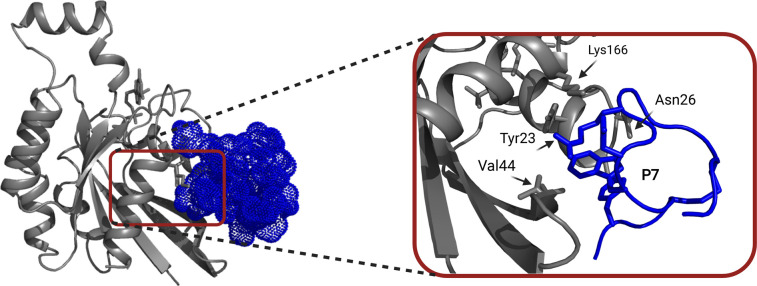
Inhibition of Cdc42-effector interactions. Cartoon of a HADDOCK model generated using NMR chemical shift titration mapping. The P7 peptide inhibitor is localized to an orthosteric binding site of Cdc42 involving key interactions of the peptide with Cdc42 residues Thr25, Asn26, Val44 and Lys166. The peptide inhibitor P7 is shown in dark blue with Cdc42 in grey (PDB 6R28).

Targeting small G protein-effector interfaces for therapeutic purposes has long been a goal in the field but protein–protein interfaces are recognized as challenging targets for small molecules [49]. A different approach, undertaken in our own laboratory, seeks to engineer peptide inhibitors of Cdc42-effector interfaces (Figure 1). This approach exploits the selectivity of the effectors themselves for their target. We selected Cdc42 binding peptides from a CIS display screen and engineered second generation peptides with low nanomolar affinity for Cdc42. NMR chemical shift mapping showed that the matured peptides bind away from the switch regions (Figure 5) but are likely to be orthosteric, clashing with binding of a selection of Cdc42 effectors, including ACK, WASP and PAK1. Binding data revealed a relatively small difference in binding between Cdc42·GDP and Cdc42·GTP, suggesting that the peptide inhibitors target both nucleotide forms of Cdc42 indiscriminately. In cell lines harbouring mutant K-Ras however, these Cdc42 inhibitors inhibited cell migration [50].

Over the last few decades there has been recognition of peptides as a rapidly expanding therapeutic modality occupying the space between small molecules and antibodies. The overall challenges facing peptides as therapeutics for the Ras-family proteins have been recently reviewed [51]. Progress in the introduction of non-canonical amino acids to expand the beneficial properties of peptides based on prior characterization of protein–protein interfaces [52], molecular grafting [53] and new cellular delivery methods including cell-penetrating peptides [54] and nanoparticles [55] highlight some of the recent advances addressing the issues of stability and delivery.

Additionally, new therapeutic approaches including nanobodies, target-binding fragments of monoclonal antibodies and PROTACs have the propensity to inhibit or deplete endogenous intracellular protein levels via the proteasomal degradation pathway. Whilst these approaches have not yet been applied to Cdc42, nanobodies selective for the GTP-bound form of RhoB, have been developed and as such represent a new strategy to inhibit the Rho family G-proteins [56].

#### Direct targeting of Cdc42 effectors

The downstream effectors of Cdc42 present a number of attractive targets for therapeutic design. The Cdc42 kinase effectors PAK1, PAK2, PAK4, MRCKα, MRCKβ and ACK have all been directly targeted, consistent with cancer mutational data indicating that these proteins show the highest incidence of alterations amongst the Cdc42 effectors [9]. However, alongside their druggable qualities they suffer from the same issues of specificity and resistance that have been widely encountered in targeting kinases [57].

**Figure 6.**
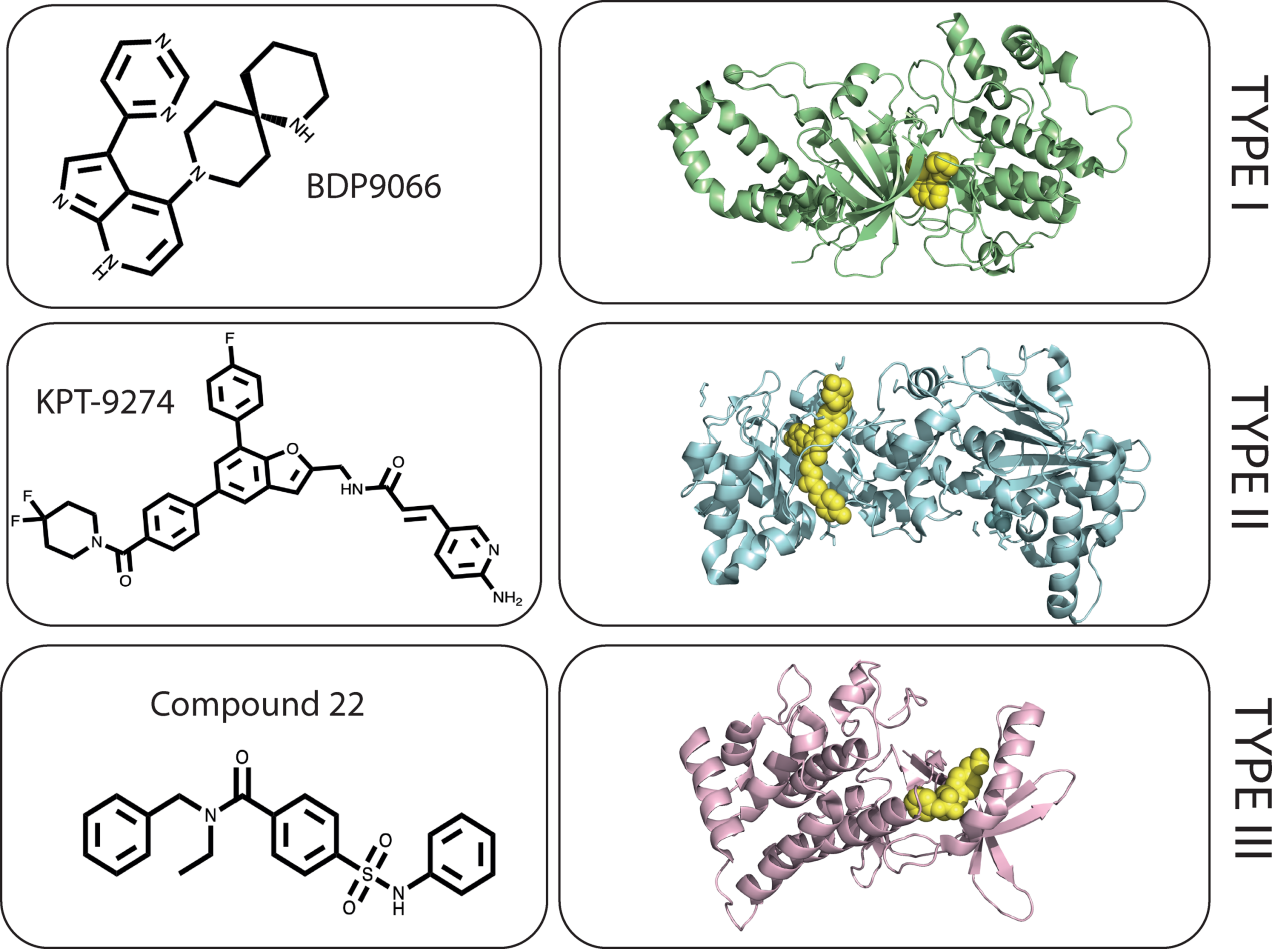
Kinase inhibitors of Cdc42 effectors. Examples of a type I kinase inhibitor of MRCK, a type II dual kinase inhibitor of PAK4/NAMPT and a type III kinase inhibitor targeted at LIMK2, effector kinases which are all found downstream of Cdc42 (**A**) A co-crystal structure of type I inhibitor BDP-9066 bound to MRCKβ (PDB:5OTF) (**B**) A co-crystal structure (PDB:5NSD) of NAMPT dimer with dual PAK4/NAMPT type II inhibitor KPT-9274 shown as yellow spheres (**C**) Type III inhibitor, Compound 22 bound in a hydrophobic binding pocket of LIMK2 (PDB:4TPT) when the DFG activation loop motif is in an inactive conformation. The carbonyl of the amide contacts backbone NH of Asp469 of the DFG motif whilst the sulfonamide carbonyl forms a hydrogen bond interaction with Arg474 adjacent to the DFG motif.

Issues in achieving selectivity for the kinases downstream of Cdc42 mainly arise from the high degree of structural conservation in the ATP binding site, where most kinases share a conserved Asp-Phe-Gly (DFG) motif capable of adopting two different conformations [58]. Type I inhibitors inhibit the kinase in the ‘DFG-in’ active conformation and compete with ATP, making critical hydrogen bonds with the kinase hinge region. Type II inhibitors bind and stabilize a kinase in its inactive ‘DFG-out’ conformation, occupying both the ATP-binding site and a hydrophobic allosteric pocket formed in the inactive conformation. Allosteric Type III and IV inhibitors represent potentially attractive alternatives to achieve kinase specificity. Type III inhibitors bind at a site adjacent to the ATP pocket without contacting the hinge region and in a hydrophobic pocket formed in the DFG-out conformation. Type IV inhibitors bind at a distinctly different allosteric site, away from the site of catalysis. Types I, II and III inhibitors have been described for Cdc42 effector kinases (Figure 6).

The PAK family have been the objective for a number of campaigns but the development of PAK inhibitors has been hampered by kinase specificity issues and toxicity due to the ubiquitous nature of their expression. A flexible ATP binding cleft has been reported in the PAK proteins [59], which may also partly explain the difficulties in developing specific type I inhibitors. PF-3758309 binds in the ATP binding site of PAK4, making hydrogen bond interactions with the hinge region and a critical charge-charge interaction with Asp458 of the DFG motif via its dimethylamine group [60]. However, PF-3758309 recently failed phase I clinical trials on the basis of a lack of dose-dependent efficacy as it was found to be a substrate for multi-drug transporter P-glycoprotein [61].

A Type II allosteric modulator of PAK4, KPT-9274, is currently in a Phase I clinical trial for advanced solid malignancies and non-Hodgkin's lymphoma (NCT02702492). KPT-9274 is a dual inhibitor of both PAK4 and nicotinamide phosphoribosyl transferase (NAMPT), a metabolic enzyme catalyzing the initial step in the biosynthetic pathway of NAD from nicotinamide [62–64]. Whilst KPT analogues were found to interact with PAK4 by chemical proteomics, inhibition of NAD^+^ biosynthesis was also reported [62]. A CRISPR-induced drug resistance screen revealed NAMPT to be the primary target for KPT-9274 [64]. NAD is an essential metabolite for proliferation and tumours have a reliance on the cofactor for rapid proliferation [65]. Dual inhibition of NAMPT and PAK4 signalling has therapeutic application in a number of cancers due to synergism [63,64,66]. KPT-924 makes pi-stacking interactions with Phe193 of the DFG motif and a Tyr of NAMPT and whilst no structure of it bound to PAK4 is available it is assumed to bind at a similar location on PAK4. Dual inhibition at lower levels of two pathways may have less associated toxicity than inhibition of a single pathway target [63]. The ability of KPT-9274 to also inhibit kinase-independent functions of PAK4 may equally result in advantageous therapeutic properties over the type I inhibitor PF-3758309. In addition to the molecules which have progressed to phase I clinical trials, a number of other inhibitors have been developed for PAK family proteins including some pan-PAK inhibitors, these have been well-reviewed elsewhere [67,68].

Progress has also been made in targeting other Cdc42 kinase effectors, for example, the small molecule inhibitors of MRCKα and MRCKβ, BDP8900 and BDP9066 (Figure 1). Structure-guided fragment elaboration identified BDP8900 and BDP9066 as more potent binders of the MRCK ATP-binding site than the previously developed BDP5290 [69]. In a screen including 750 human cancer cell lines across 40 cancer types, haematologic cancers were identified as most susceptible to BDP9066. Evidence for therapeutic efficacy in skin cancer has also been shown: topical treatment with BDP9066 reduced skin papilloma outgrowth in a model of murine squamous cell carcinoma [70]. Overall, the development of the more potent and selective molecules should enable further dissection of the potential for MRCK inhibition in cancer. There are also several lines of evidence suggesting that dual inhibition of the Rho effector ROCK with MRCK may show increased efficacy [71].

ACK inhibitors have not made the same progress as inhibitors for the other kinases downstream of Cdc42 and all of the reported small molecule ACK inhibitors target the ATP-binding site [72]. The best characterized and experimentally most widely used ACK inhibitor is AIM-100. Unsurprisingly a number of molecules originally designed as tools targeting other kinases also inhibit ACK as they target the highly conserved ATP site [72]. For example, NVP-TAE684, an inhibitor of the oncogenic tyrosine kinase, NPM-ALK [73] also binds ACK with a *K*_d_ of 2 nM [74]. However, NVP-TAE684 has not progressed due to potential toxicity issues.

PAK and MRCK pathways converge further downstream on LIM kinases. Obtaining selectivity between LIMK1 and LIMK2 with Type I and II inhibitors has posed a significant challenge, due to their 71% kinase domain sequence identity [75]. However several compounds have been developed, with high binding affinities and desirable pharmacokinetic properties [76]. A number of type I kinase inhibitors and allosteric compounds have been developed for both LIMK1 and LIMK2. A dual inhibitor of LIMK2 and ROCK (LIMK2, IC_50_ 7.5 nM), LX7101, has completed a Phase 1/2a study in primary open-angle glaucoma and ocular hypertension (NCT01528111) but the results are yet to be made available. The first Type III inhibitor reported for LIMK2, a sulfonamide compound, ‘compound 22’, binds in a hydrophobic pocket formed in the in inactive DFG-out conformation away from the hinge region and is ATP non-competitive [77]. It was shown to have good selectivity for LIMK2 (IC_50_ = 39 nM) over LIMK1 (IC_50_ = 3.2 µM). Despite the development of different classes of kinase inhibitors, the analysis of LIMK inhibitors as anticancer therapeutics in clinical trials is still unexplored.

### Cdc42 membrane attachment inhibitors

An alternative approach to attacking downstream of Cdc42, is to target membrane localization of the GTPase, another strategy considered early on in the pursuit of Ras targeted therapeutics. Cdc42 is modified at its C-terminus by a prenyl group and inhibition of this modification has been explored. Prenyl transfer is catalyzed by type 1 geranylgeranyl transferases (GGTase I) and several GGTase I inhibitors have been developed. Additionally, the use of statins, which inhibit the synthesis of the substrate of GGTase I, geranylgeranyl pyrophosphate, have also been investigated. However, the wide range of proteins that are substrates for the geranylgeranyl transferases leads to a large number of affected protein functions, generating toxic side effects and explaining why, to date, no GGTase inhibitors have been approved for clinical use [78].

Secramine A, an analogue of the natural product, Galanthamine, acts by inhibiting membrane association of geranylgeranylated Cdc42 [79]. Various analyses determined that the inhibitory effects of Secramine A are dependent on RhoGDI-1, a molecular chaperone that shuttles Rho family GTPases between the cytosol and target membranes. Secramine A prevented the return of Cdc42 to PIP_2_ liposomes and the subsequent activation of Cdc42, and it inhibited *in vitro* assembly of PIP_2_ liposome-stimulated actin polymerization upstream of Arp2/3 complex activation. Other phenotypic effects regulated by Cdc42-dependent pathways were also documented, with a significant inhibition of reorientation of the Golgi apparatus observed in cells treated with Secramine A [79]. Unanswered mechanistic questions include the precise contribution of RhoGDI-1 to the effects of Secramine A. It is unknown if, for example, Secramine A binds at the interface of Cdc42-RhoGDI-1 to stabilize the complex and therefore decrease the pool of available Cdc42 for activation and effector signalling.

## Conclusions and future perspectives

It is a pertinent time to explore the range of approaches that have been applied to target Cdc42 therapeutically. The challenges of inhibiting Cdc42 can be broadly separated into two themes: structural and biological. Broadly, from a structural perspective, the challenges are achieving potency and selectivity between overlapping binding sites for downstream effectors and regulatory proteins. The challenge of specifically inhibiting Cdc42 interactions is underpinned by the high homology within the Rho family GTPases and in the wider Ras superfamily. For Cdc42 itself the overlap between different effector and regulatory binding partners makes selective inhibition difficult to achieve. However, therapeutic approaches based on for example, previously unappreciated binding pockets and orthosteric binding sites, such as that identified for anti-Cdc42 peptide inhibitor P7, which likely selectively inhibits effector binding without inhibiting GEF, GAP and GDI binding, could address the challenge of selective inhibition. Additionally, energetic analysis of the binding interfaces has identified potential residue specificity hotspots to target [80,81] indicating progress could still be made.

Overall, new therapeutic approaches based on, for example, previously unappreciated binding pockets, along with new modalities such as peptides, PROTACs and nanobodies likely will have relevance for targeting this classical member of the Rho family GTPases. Whilst the centrality of Cdc42 to actin cytoskeleton dynamics is evident, there is still a need to establish the importance of the role of Cdc42 in regulating metastasis and invasion and the functional consequences of the Cdc42 mutations that have been identified in the cancer genome projects [9]. However it is reasonable to assume inhibiting Cdc42 will be effective in inhibiting invasion and metastatic dissemination, given that it has been linked to early events of metastasis and shown to regulate both forms of metastatic cell motility [9]. Additionally, there is potential to exploit the other recurrently altered molecular targets downstream of Cdc42 and the interfaces between their downstream effectors, GEFs, GAPs and GDIs.

## Perspectives

- ***Importance of the field:*** Cdc42 lies downstream of the master regulator Ras and is crucial for cell transformation by Ras, making Cdc42 and the pathways it controls key targets for oncology therapeutics. Multiple strategies have been applied to the targeting of Cdc42 in attempting to achieve both specificity and efficacy for this classical member of the Rho family, its regulatory proteins and downstream effectors.
- ***Current thinking:*** Many of the small molecules which have been developed are pan or dual inhibitors of both Cdc42 and its very close relative, Rac1. New therapeutic approaches taken to Cdc42 inhibition include an irreversible covalent pan-Rho family inhibitor molecule and an anti-Cdc42 peptide inhibitor.
- ***Future directions:*** Assessing the individual biological effects due to Cdc42 inhibition and therefore the contribution of Cdc42 to regulating migration and invasion in cancer requires further advances in selectivity. However, there are also potential applications for dual inhibition of Rac1/Cdc42. New modalities and strategies of inhibition currently in development will likely contribute to further advances in targeting Cdc42 successfully.

## Open Access

Open access for this article was enabled by the participation of University of Cambridge in an all-inclusive *Read & Publish* pilot with Portland Press and the Biochemical Society under a transformative agreement with JISC.

## Abbreviations

ACK: Activated Cdc42-associated kinase
ATP: adenine nucleotide triphosphate
Cdc42: cell division control protein 42
GAP: GTPase activating protein
GDP: guanine nucleotide diphosphate
GEF: guanine nucleotide exchange factor
GGTase: geranygeranyl transferase
GTP: guanine nucleotide triphosphate
LIMK: LIM domain protein kinase
MRCK: myotonic dystrophy kinase-related Cdc42-binding kinases
NAMPT: nicotinamide phosphoribosyl transferase
PAK: p21 activated kinase
PROTAC: proteolysis targeting chimera
Rac1: Ras-related C3 botulinum toxin substrate 1
RhoA: Ras homology family member A
RhoGDI: guanine dissociation inhibitor for Rho family G proteins
WASP: Wiskott Aldrich Syndrome proteins
Arp2/3: Actin Related Protein 2/3 complex
PIP_2_: phospho-4,5-bisphosphate
